# Intraperitoneal Liposarcoma: A Case Report and Literature Review of a Rare Entity

**DOI:** 10.7759/cureus.59244

**Published:** 2024-04-28

**Authors:** Abdullah K AlBathi, Yahya M Mashhor, Abdullah A Muharib, Abdulaziz A Altawili

**Affiliations:** 1 Radiology, King Fahad Medical City, Riyadh, SAU; 2 Radiology, Altakassusi Alliance Medical, Riyadh, SAU

**Keywords:** mdm2, intra-peritoneal, liposarcoma, recurrent sarcoma, dedifferentiated liposarcoma

## Abstract

Liposarcoma is a rare soft-tissue neoplasm originating from adipocytes. The exact cause of liposarcoma is unknown and symptoms vary depending on the tumor’s location. A 49-year-old man presented to the emergency room complaining of epigastric pain radiating to the back and right upper quadrant. Cross-sectional imaging revealed a large upper abdominal mass that was thought to be a gastrointestinal stromal tumor (GIST) arising from the duodenum at first. The patient underwent en-bloc resection of the mass and was planned for adjuvant chemotherapy. Subsequently, multiple tissue samples were examined, leading to the final diagnosis of de-differentiated liposarcoma. The patient eventually developed multiple recurrences and was subjected to re-resection surgeries and three different chemotherapy regimens. Given the rarity of the disease, no standardized therapy plan is available, highlighting the need for more case reports/series and trials to broaden our understanding of this disease.

## Introduction

Liposarcoma is a rare soft-tissue neoplasm originating from adipocytes. The World Health Organization reports that there are five histopathological types of liposarcoma: well-differentiated (WDLPS), dedifferentiated (DDLPS), mixed (MLPS), myxoid/round cell (RC), and undifferentiated pleomorphic (UPS) [1]. The exact cause of liposarcoma is unknown, but it is thought to be caused by mutations in adipocyte DNA. Liposarcomas are more common in adults than in children and is most often diagnosed in people over the age of 50 years.

The symptoms of liposarcomas vary depending on the location of the tumor. Patients with intra-abdominal lesions or masses are most likely to present with asymptomatic abdominal fullness or pain. They can occur anywhere in the body, but most commonly occur in the lower limbs, followed by the retroperitoneum. Intraperitoneal origin, however, is an extremely rare location with unknown incidence, as it has only been mentioned in case reports. This paper presents a case of DDLPS arising from the porta hepatis that was initially misdiagnosed and complicated by multiple recurrences. It also reviews the literature on intraperitoneal liposarcoma.

## Case presentation

**Figure 1:**
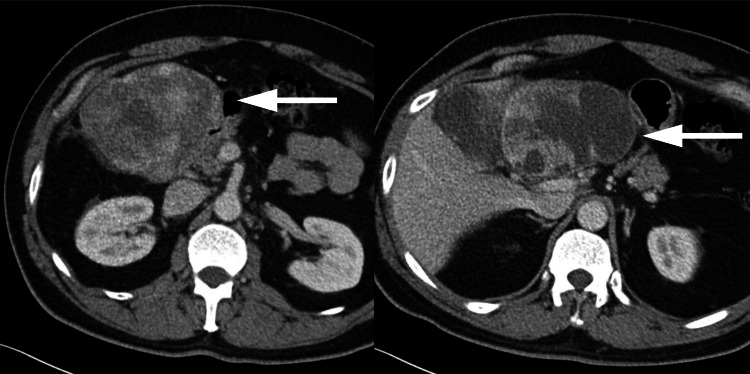
Contrast-enhanced CT images of the abdomen (axial view) CT: computerized tomography Axial contrast-enhanced CT images showing a large complex mass of cystic and solid nature (white arrows) in the paraduodenal area extending into the porta hepatis. The mass appears inseparable from the adjacent structures, including the liver, portal triad, duodenum, pancreas, and pylorus, with no definite organ or vascular invasion.

**Figure 2:**
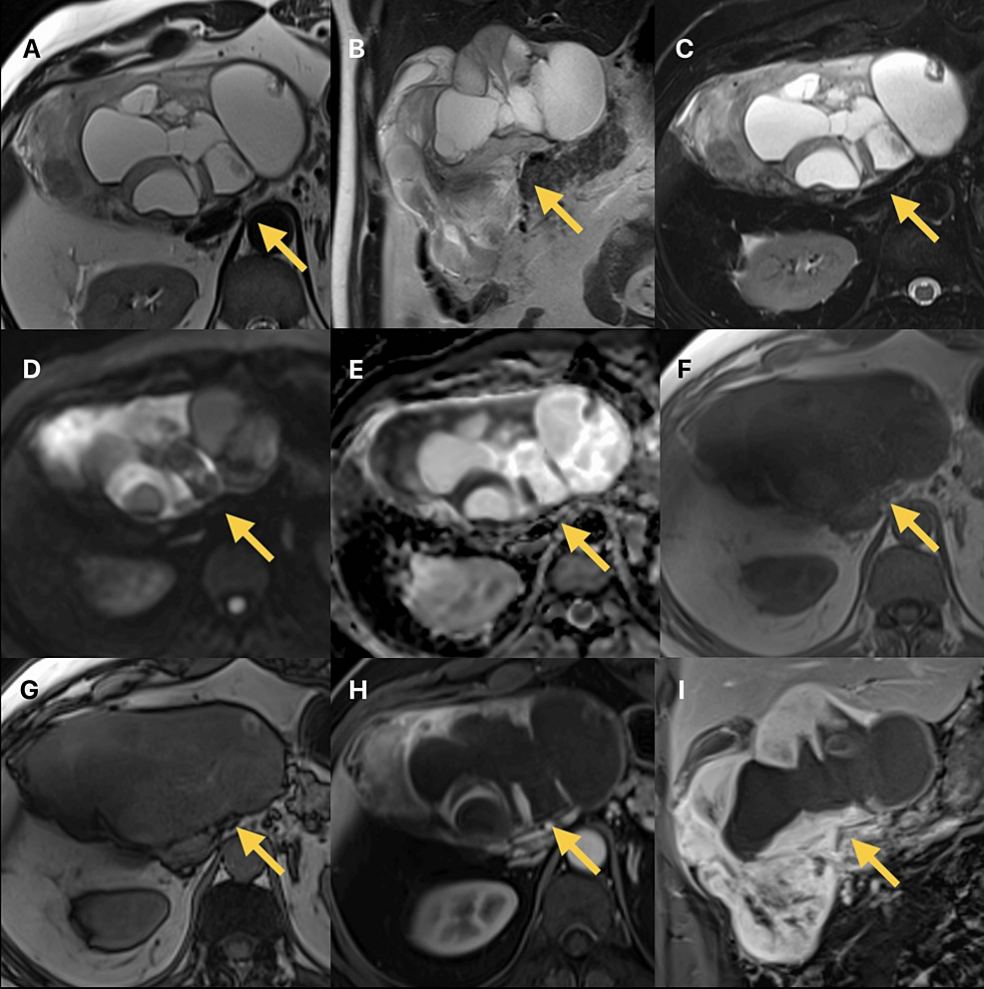
T2 weighted MR images MR: Magnetic resonance; FSE: Fast-spin echo; DWI: Diffusion-weighted imaging; ADC: Apparent diffusion coefficient. Axial (A) and coronal (B) T2 weighted MR images single shot FSE show huge complex cystic and intermediate hyperintense soft tissue mass in the paraduodenal region extending into the portahepatis. The mass appears inseparable from the duodenum and is abutting the liver and pancreas. Axial fat-suppressed T2 weighted MR image (C) shows the complex nature of the mass with cystic changes and soft tissue components that demonstrate intermediate high signal intensity. Axial DWI (D) and ADC maps (E) (b value = 800 s/mm²) showed high signal intensity on DWI and low signal intensity on the ADC maps of the soft tissue components, indicating diffusion restriction. Axial T1 gradient echo in-phase (F) and out-of-phase (G) MR images showed a predominantly low T1 signal intensity mass with areas of high signal intensity without signal drop in the out-of-phase to suggest fat components. Axial (H) and coronal (I) contrast-enhanced MR in the portal venous phase showed heterogeneous enhancement of the soft-tissue components of the mass. The yellow arrows mark the mass being described.

A 49-year-old man with no prior history of illness or surgery presented to the emergency room (ER) complaining of epigastric pain radiating to the back and right upper quadrant. The patient noticed pain a month before presentation, which increased in severity and became less responsive to analgesics. Contrast-enhanced computerized tomography (CT) (Figure 1) scanning and magnetic resonance imaging (MRI) of the abdomen (Figure 2) were done and revealed a large complex mass of cystic and solid nature in the paraduodenal area extending into the porta hepatis. With no discernible organ or vascular invasion, the mass appeared to be inseparable from the adjacent organs, including the liver, portal triad, duodenum, pancreas, and pylorus. The mass was originally thought to be a gastrointestinal stromal tumor (GIST) originating from the duodenum based on radiological features.

**Figure 3:**
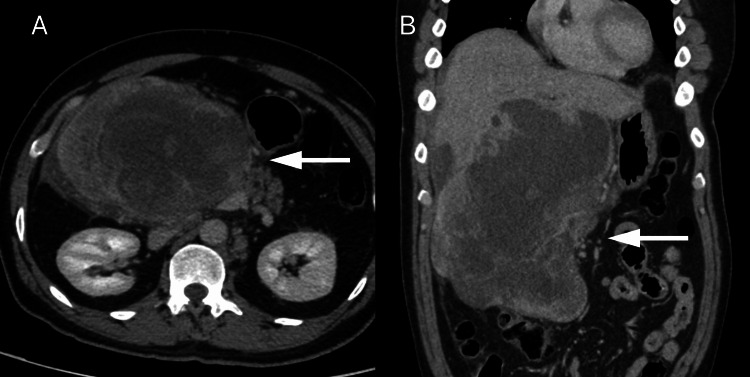
Contrast-enhanced CT images in the portal venous phase CT: computerized tomography Axial (A) and coronal (B) contrast-enhanced CT images in the portal venous phase show the huge heterogeneously enhancing complex mass measuring 20.5 x 14.8 x 11 cm (white arrows). The mass appears inseparable from the adjacent organs and compresses the common bile duct causing intrahepatic biliary duct dilatation. Enlarged retroperitoneal lymphadenopathy has also developed (not shown).

After two months only, the patient was referred to our tertiary center. A laboratory investigation, including tumor markers, was performed. The results revealed elevated levels of liver enzymes with an obstructive pattern. None of the tumor markers were positive (Table 1). A repeat CT scan of the abdomen and pelvis (Figure 3) revealed a dramatic increase in the size of the mass with obstruction of the common bile duct. No thoracic lesions were noted on the chest CT. 

**Table 1:** Laboratory workup on presentation to our tertiary center *abnormal value; ALT: Alanine aminotransferase; AST: Aspartate aminotransferase; ALP: Alkaline phosphatase; CEA: Carcinoembryonic antigen; CA 19-9: Cancer antigen 19-9.

Lab test	Result	Reference range
ALT	143 U/L *	0 - –55 U/L
AST	147 U/L *	5 - –34 U/L
ALP	313 U/L *	40 - –150 U/L
Total Bilirubin	24.2 umol/l *	3.4 - –20.5 umol/l
Direct Bilirubin	17.22 umol/l *	<8.6 umol/l
Lipase	35.9 U/L	8 - –78 U/L
Amylase	75 U/L	25 - –125 U/L
WBC	7,720/µL	3,900 - –11,000//µL
Hemoglobin	11.6 g/dl *	13.5 - –18 g/dl
CEA	1.73 µg/L	<5 µg/L
CA 19-9	4.7 u/ml	<37 u/ml
Alpha-Fetoprotein	2.2 µg/L	1.89 -– 8.78 µg/L

Percutaneous core biopsy of the mass revealed an atypical spindle cell neoplasm. First, owing to its aggressive radiological features and focal reactivity to smooth muscle actin (SMA) and desmin, leiomyosarcoma was diagnosed. Cytogenetic and immunohistochemical analyses initially ruled out DDLPS, as the samples were negative for mouse double minute 2 (MDM2) amplification, and GIST, as DOG1 and CD117 immunostaining, were negative.

After the tumor board discussion, the patient was scheduled for cytoreductive surgery and adjuvant systemic chemotherapy of doxorubicin-ifosfamide for four cycles. In addition to cholecystectomy and partial hepatectomy, an en-bloc resection of the mass was performed with the intention of a cure. Grossly, the large mass appeared lobulated and mostly cystic, with a soft tissue component extending from the liver to the right iliac fossa with attachments to the gastric wall and portal triad. Multiple tissue samples were subjected to pathological analysis. All of the resected tissues showed negative margins. MDM2 amplification was confirmed and a final diagnosis of DDLPS was made. Immunohistochemistry results were positive for S100, CD34, desmin, SMA (in spindle cells), caldesmon, CD10, and BCl2, while negative for myogenin, synaptophysin and chromogranin A, GFAP, CK, and SOX-10. 

**Figure 4:**
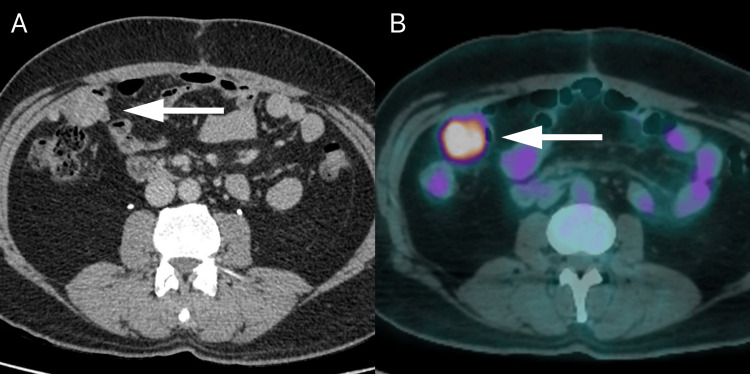
Tomography images four months after tumor resection CT: Computerized tomography; PET: Positron emission tomography. Axial (A) contrast-enhanced CT image in portal venous phase shows tumor recurrence/residual (white arrow). Axial fused fluorodeoxyglucose (18F) PET/CT image (B) shows hypermetabolic recurrent/residual mass (white arrow).

Follow-up imaging (Figure 4A) four months later, after receiving only three cycles of chemotherapy, showed the interval development of a small lesion in the right paracolic gutter, consistent with disease recurrence/residual. A positron emission tomography fluorodeoxyglucose (18F) scan of the whole body (Figure 4B) showed avid uptake by the lesion, heralding disease involvement. No bone involvement or distant metastases were observed.

Two months later, CT of the abdomen, performed as part of the pre-operative planning for re-resection (not shown), showed rapid growth and aggressive nature of the mass. Two large masses and multiple peritoneal deposits were observed. The patient eventually underwent another resection of the large lesions with peritonectomy.

**Figure 5:**
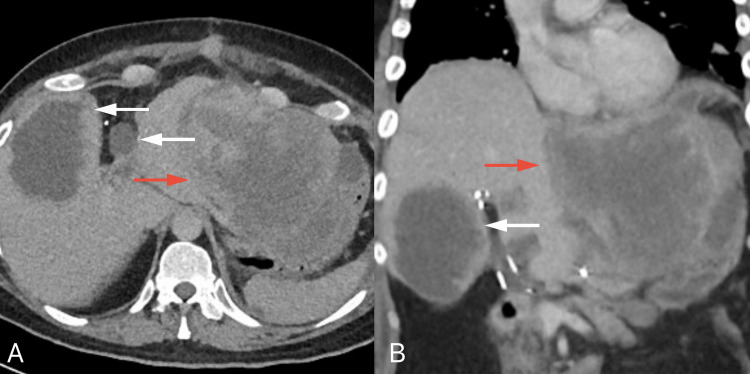
CT scans images a month after the second cytoreductive surgery CT: Computerized tomography Axial (A) and coronal (B) CT scans done a month after the second cytoreductive surgery showed a large internal hematoma with features of active venous bleeding (red arrows). Multiple peritoneal nodules showed an increase in size; the subhepatic and gastrohepatic nodules are shown here (white arrows).

A month later post-re-resection, in a follow-up office visit, an alarming decrease in hemoglobin, from 12 to 7.9 g/dL, was noticed. The patient was sent to the ER, where a CT scan of the abdomen was obtained, which revealed tumor recurrence with interval enlargement and an internal hematoma with active hemorrhage (Figure 5). A third round of surgery was deemed. After admission, the patient underwent an en-bloc resection with partial hepatectomy. Histopathological examination revealed peritoneal and hepatic lesions consistent with those of DDLPS. A follow-up CT scan revealed an increase in the size of the previously noted peritoneal deposits (not shown). During follow-up with the oncology team, the patient was planned to start a second-line systemic chemotherapy regimen comprising gemcitabine and dacarbazine. The chemotherapy regimen was delayed for three weeks, as the patient was having constant drops in hemoglobin. After stabilization and completion of four cycles of the new regimen, a follow-up CT scan of the abdomen (not shown) revealed disease progression. Consequently, the decision was made to switch the patient to a palliative systemic chemotherapy regimen involving eribulin. Additionally, a 'do not resuscitate' order was issued, and the patient was referred to palliative care for the management of abdominal pain.

## Discussion

DDLPS typically displays high-grade morphology and metastases in 15-20% of cases [1,2]. Henrick et al. studied 155 cases of DDLPS, the median age of which was 61.5 years and ranged from 21 to 92 years. Tumors most commonly occur in the retroperitoneum, followed by the extremities and trunk. Most DDLPSs present as de-novo lesions [3]. 

We conducted an advanced search in the MEDLINE database and another search using the MeSH terms “Liposarcoma” and “Intraperitoneal Neoplasms.” After reviewing the literature, we found that fewer than 90 cases of liposarcomas originating from the intraperitoneal compartment of the abdomen have been reported. Sixty-eight patients [4-67] (Table 2) with sufficient information were included in this study (including the present case). 

**Table 2:** Results of the literature review of intraperitoneal liposarcomas M: Male, F: Female, RCLPS: Round cell liposarcoma, WDLPS: well-differentiated liposarcoma, DDLPS: de-differentiated liposarcoma, MLPS: mixed liposarcoma,  UPLPS: un-differentiated pleomorphic liposarcoma, N/A: information was not available.

Author	Age & Sex	Location	Histo-pathology	Treatment	Outcome
Hightower et al. [4]	11 M	Greater omentum	RCLPS	Resection	N/A
Rosato et al. [5]	55 M	Mesentery	WDLPS	Resection	No recurrence after 12 years.
Rosato et al. [5]	55 M	Mesentery	RCLPS	Resection	Recurrences after 5 and 14 years.
Rosato et al. [5]	52 M	Lesser omentum	WDLPS	Resection	No recurrence after 20 years
Nohara et al. [6]	65 M	Multifocal (intraperitoneal and retroperitoneal space)	RCLPS	Resection	Recurrence in 4 months. Patient deceased after 7 months post-operation.
De et al. [7]	45 M	Omentum	RCLPS	Resection	Deceased after 9 months post-operation.
Stout et al. [8]	60 F	Omentum with peritoneal deposits and metastases to the liver	RCLPS	Biopsy	Deceased after two days post-biopsy.
Imai et al. [9]	55 F	Greater omentum with peritoneal spread	RCLPS	Resection	Deceased within 1 month post operation.
Garg et al. [10]	47 F	Mesentery	DDLPS	Resection + adjuvant chemotherapy (doxorubicin, cisplatin, and ifosfamide)	Deceased after the first cycle of chemotherapy due to sepsis.
Garg et al. [10]	63 M	Mesentery	DDLPS	Resection + adjuvant chemo-radiotherapy	Recurrence after 1 year
Alecu et al. [11]	56 M	Lesser omentum	DDLPS	Resection + omentectomy + partial gastrectomy	N/A
Cai et al. [12]	37 F	Disseminated intraperitoneal and greater omentum	DDLPS	Resection + omentectomy + adjuvant doxorubicin chemotherapy	N/A
Sato et al. [13]	72 M	Multifocal (intraperitoneal cavity attached to stomach, retroperitoneum)	WDLPS	Resection	No recurrence after 12 months of follow-up.
Soufi et al. [14]	65 F	Greater omentum	DDLPS	Resection + omentectomy + appendectomy + adjuvant doxorubicin chemotherapy	No recurrence after 18 months of follow-up.
Constantinoiu et al. [15]	73 M	Sigmoid mesocolon	DDLPS	Resection	No recurrence after 6 months of follow-up.
Miwa et al. [16]	51 M	Greater omentum	DDLPS	Resection + Partial sigmoid colon and bladder serosa resection	No recurrence after 10 months of follow-up.
Atram et al. [17]	61 F	Greater omentum with intraperitoneal metastases	DDLPS	Total abdominal hysterectomy, bilateral salpingo-oophorectomy, with debulking of omental mass, and multiple metastatic deposits on the peritoneum + adjuvant doxorubicin and ifosfamide chemotherapy	N/A
Dhakal et al. [18]	56 M	Small bowel mesentery	DDLPS	Resection + partial ileal resection	N/A
Yemez et al. [19]	72 M	Intraperitoneal and left inguinal canal	DDLPS	Resection	N/A
Okajima et al. [20]	54 F	Omental	RCLPS	Resection	No recurrence after 10 months of follow-up.
Tsutsumi et al. [21]	83 M	Omentum	RCLPS	Resection	No recurrence after two years of follow-up.
Fotiadis et al. [22]	64 F	Omentum	RCLPS	Resection	Two recurrences within nine years.
Alameda et al. [23]	25 F	Omentum	RCLPS	NA	NA
Milic et al. [24]	52 F	Omentum reaching the inguinal hernia	RCLPS	Resection + herniotomy	No recurrence after 3.5 years of follow-up.
McAvoy et al. [25]	65 M	Omentum	RCLPS	Resection + partial gastrectomy + adjuvant doxorubicin chemotherapy.	N/A
Burgohain et al. [26]	32 F	Small intestinal mesentery	WDLPS	Resection	N/A
Karaman et al. [27]	62 M	Mesentery	DDLPS	Resection + adjuvant radiotherapy	No recurrence after 15 months of follow-up.
Tsoukalas et al. [28]	47 F	Mesentery	MLPS	Resection + adjuvant chemotherapy	N/A
Murata et al. [29]	46 M	Intraperitoneal and retroperitoneal masses	RCLPS	Resection + adjuvant chemotherapy	No recurrence after nine months of follow-up.
Kim and Jee [30]	60 M	Mesentery	RCLPS	Resection + adjuvant chemotherapy	N/A
Meloni et al. [31]	34 M	Greater omentum	WDLPS	Resection	No recurrence in five years of follow-up.
Amato et al. [32]	75 F	Sigmoid mesocolon	WDLPS	Resection	No recurrence after two years of follow-up.
Choi et al. [33]	73 M	Small bowel Mesentery	UPLPS	Resection + partial ileal resection + adjuvant chemotherapy	No recurrence after 25 months follow up..
Edakuni et al. [34]	NA	Transverse mesocolon	RCLPS	Resection	No recurrence after 17 months of follow-up.
Takeda et al. [35]	71 M	Transverse and ascending mesocolon	DDLPS	Resection + partial pancreatectomy	Recurrence after six months.
Winn et al. [36]	59 M	Sigmoid mesocolon	DDLPS	Left hemicolectomy + Splenectomy	Recurrence after two months.
Jukić et al. [37]	77 M	Mesentery	MLPS	Resection	N/A
Gupta et al. [38]	45 M	Mesentery	WDLPS	Resection	Recurrence after five years.
Korukluoglu et al. [39]	61 M	Bilateral mesentery	DDLPS	N/A	N/A
Park et al. [40]	47 M	Ascending colon mesentery	DDLPS	Resection + hemicolectomy + adjuvant doxorubicin, ifosfamide, and mesna chemotherapy	No recurrence after 21 months of follow-up.
Hashimoto et al. [41]	60 F	Greater omentum	WDLPS	Resection	No recurrence after nine months of follow-up.
Niromanesh et al. [42]	57 F	Anterior to the peritoneum in the left lower part of the abdomen	WDLPS	Resection + adjuvant chemotherapy	No recurrence after five months of follow-up.
Vats et al. [43]	36 F	Mesentery	DDLPS	Resection + partial of jejunectomy + adjuvant doxorubicin, dacarbazine and ifosfamide) chemotherapy	No recurrence after 12 months of follow-up.
Meher et al. [44]	62 M	Small bowel mesentery	DDLPS	Resection + Segmental resection of the small bowel	No recurrence after 10 months of follow-up.
Matsuo et al. [45]	70 M	Small bowel mesentery	DDLPS	Resection + Segmental resection of the small bowel	No recurrence after five years of follow-up.
Mori et al. [46]	71 M	Small bowel mesentery	DDLPS	Resection + ileocecal and sigmoid colon resection	17 day post-resection recurrence - eribulin given was not effective thus pazopanib was given which lead to shrinking of the size of the mass.
Hirakoba et al. [47]	65 F	Small bowel mesentery	WDLPS	Resection	N/A
Cerullo et al. [48]	55 M	Mesentery	WDLPS sclerosing type	Resection	N/A
Khan et al. [49]	52 M	Mesentery	WDLPS	Resection	No recurrence after five years of follow-up.
Khan et al. [50]	55 M	Two mesenteric masses	WDLPS	Resection	N/A
Khanduri et al. [51]	55 M	Jejunal mesentery	DDLPS	Resection + adjuvant ifosfamide and doxorubicin chemotherapy	No recurrence after two months of follow-up.
Poilluci et al. [52]	43 M	Mesentery	WDLPS	Resection + small bowel resection	N/A
Poilluci et al. [52]	60 M	Mesentery	WDLPS	Resection + small bowel resection	N/A
Mokfi et al. [53]	69 F	Mesentery	RCLPS	Resection + left nephrectomy	No recurrence after six months of follow-up.
Ahire et al. [54]	42 M	Mesentery of the jejunum	DDLPS	Resection + adjuvant doxorubicin, ifosfamide, and mesna chemotherapy	No recurrence after six months of follow-up.
Yuri et al. [55]	73 M	Duodenal mesentery	WDLPS	Resection	No recurrence after six months of follow-up.
Calo et al. [56]	N/A	Mesentery	WDLPS	Resection	No recurrence after 33 months of follow-up.
Shen et al. [57]	49 F	Sigmoid mesocolon + two tumors in the pelvis	RCLPS	Resection + partial colectomy	No recurrence after 17 months of follow-up.
Eltweri et al. [58]	41 F	Mesocolon	RCLPS	Resection + right hemicolectomy	Recurrence after six years.
Ngatchou Djomo et al. [59]	64 F	Right Mesocolon	WDLPS	Resection + right hemicolectomy	No recurrence after 12 months of follow-up.
Zhang et al. [60]	65 M	Mesentery	DDLPS	Resection + right hemicolectomy	No recurrence after six months of follow-up.
Jain et al. [61]	50 M	Mesentery	UPLPS	Resection + partial jejunectomy	N/A
Grifasi et al. [62]	59 M	Mesentery	DDLPS	Resection	Recurrence after five months.
Liu et al. [63]	59 F	Mesentery	DDLPS	Resection	Recurrence after nine months.
Suzuki et al. [64]	53 M	Ascending mesocolon	WDLPS sclerosing type	Resection + right hemicolectomy	N/A
Duman et al. [65]	45 M	Mesentery	DDLPS	Resection	N/A
Rajendran et al. [66]	47 M	Greater curvature of the stomach	WDLPS	Resection	N/A
Presented case	49 M	Porta-hepatis	DDLPS	Resection + partial hepatectomy + cholecystectomy + adjuvant chemotherapy	Three recurrences + residuals within one year, underwent re-resection for two more times.

Of the 68 cases of intraperitoneal liposarcomas, 26 (38.2%) were of the dedifferentiated subtype, 20 (29.4%) were of the well-differentiated subtype, 18 (26.4%) were of the myxoid/round cell subtype, two (2.9%) were of the pleomorphic subtype, two (2.9%) were of the mixed subtype. A male predilection was noted, as 45 (66.2%) of the included cases were males, and 21 (30.8%) were females; i.e., the age and gender were not mentioned in two cases. The average age of the patients affected by intraperitoneal liposarcomas was 55.9 years. The youngest patient was an 11-year-old boy [4].

Recurrence occurred in 12 patients, including the present case. The mean for follow-up in reports with recurrence is 28.5 months (range 0.5-168 months), while the mean for follow-up in reports without recurrence is 27.7 months (range 1-240 months). Rosato et al. included three patients with intraperitoneal liposarcoma; their study had the longest follow-up period of 20 years, during which the patient remained disease-free [5]. The same study reports a recurrence of intraperitoneal liposarcoma after 14 years [5]. Five studies report the expiration of patients during the postoperative period because of the extensiveness of the disease, suppression of the immune system from chemotherapy, and/or postoperative complications [6-10]. Twenty-two of the case reports included did not mention follow-up.

It is noted that recurrence more often occurs with lesions that are in “risky” locations, such as those that are near organs or major vessels, limiting the ability of resection with clear margins. However, negative margins do not guarantee any recurrence. Our patient had negative margins in the first resection surgery and still developed local recurrence along with new peritoneal disease. It is also worth mentioning that, in our case, the patient was first diagnosed with leiomyosarcoma because MDM2 amplification was negative in the core biopsy. However, resected tissue samples were positive for MDM2 amplification, leading to the diagnosis of DDLPS.

According to the STRASS trial, which was conducted to assess the effectiveness of radiotherapy for retroperitoneal sarcomas, pre-operative radiotherapy should not be considered the standard of care for retroperitoneal sarcomas [67]. This is also likely true for intraperitoneal sarcomas; however, no trial was performed on such entities. Insufficient evidence supports hyperthermic intra-peritoneal chemotherapy (HIPEC) or early post-operative intra-peritoneal chemotherapy (EPIC) for peritoneal sarcomatosis [68]. Chemotherapy for dedifferentiated liposarcomas has shown clinical benefits, but overall survival remains poor [69]. The most effective systemic chemotherapy regimen for soft tissue sarcomas involving the extremities was the combination of doxorubicin and ifosfamide [70].

Intraperitoneal liposarcomas are rare and require no standard care. They have multiple sites of origin from within the intraperitoneal compartment, including the small bowel mesentery, greater omentum, mesocolon, and porta hepatis. The rarity of the disease is an obstacle to a better understanding and conduction of formal clinical trials.

## Conclusions

Intraperitoneal DDLPS is extremely rare and might be elusive to diagnose. We present a case of aggressive intraperitoneal DDLPS with multiple recurrences that was treated three times with cytoreductive surgery and two adjuvant chemotherapy regimens that were not able to halt disease progression and was started on the third regimen. Given the rarity of the disease, no standardized therapy plan is available, highlighting the need for more case reports/series and trials to broaden our understanding of the disease and its treatment.
